# The correlation between pro- and anti-inflammatory cytokines and anti-spike IgG antibody responses induced by the SARS-CoV-2 coronavirus vaccine

**DOI:** 10.3389/ebm.2025.10849

**Published:** 2026-01-14

**Authors:** Mustafa Abdulkareem Salman, Thowiba Yousif Jameel, Abdurrahman Ayvaz, Ahmed Rushdi Abdullah

**Affiliations:** 1 Department of Biology, Graduate School of Natural and Applied Science, Erciyes University, Kayseri, Türkiye; 2 Department of Anatomy, College of Medicine, University of Diyala, Diyala, Iraq; 3 Department of Biology, Faculty of Science, Tikrit University, Tikrit, Iraq; 4 Department of Biology, Faculty of Science, Erciyes University, Kayseri, Türkiye; 5 Head of the Microbiology Department, Medical College, Aliraqia University, Baghdad, Iraq

**Keywords:** anti-inflammatory, anti-spike IgG, BNT162b2 mRNA, Pfizer-BioNTech, pro-inflammatory

## Abstract

Even with the development of the Pfizer-BioNTech BNT162b2 vaccine, which provides protection against COVID-19 and demonstrates high efficacy in generating immune responses, the complexities of the dynamics linking pro- and anti-inflammatory cytokine profiles with anti-spike IgG production remain unclear. The study aims to elucidate these immune dynamics after vaccination. This prospective cohort research was done at the University of Diyala from January 2022 to January 2023, evaluating the immunological response to the Pfizer-BNT162b2 mRNA vaccine in 180 healthy students. Pro- and anti-inflammatory cytokines and anti-spike IgG antibodies were measured before vaccination, 1 month after the second dose, and 4 months after the second dose. Biomarkers were analyzed via ELISA and CRP assays. The study involved 180 healthy participants (80 males, 100 females; median age, 21 years; BMI, 25.7 kg/m^2^). After the first Pfizer-BNT162b2 vaccine dose, the level of anti-spike IgG increased by 330-fold, and the levels of pro- and anti-inflammatory markers, such as IL-1β, IL-10, and CRP, increased significantly. Four months after the second dose, anti-spike IgG levels were 136-fold above baseline. Significant correlations emerged between cytokine and IgG levels, with anti-spike IgG/IL-10 ratios elevated and sustained over the long term. Pfizer-BNT162b2 vaccine elicits a significant immune response associated with changes in pro-inflammatory cytokines, and the interaction between these cytokines and anti-spike IgG suggests a potential role for immune regulation in enhancing humoral immunity. Based on these findings, the IgG/IL-17 ratio may serve as a viable exploratory biomarker for assessing short- and medium-term vaccination efficacy.

## Impact statement

This study investigates the paradoxical interplay between pro- and anti-inflammatory cytokines and anti-spike IgG responses following administration of the Pfizer-BioNTech BNT162b2 vaccine. A prospective cohort of 180 healthy, unvaccinated medical students (median age: 21 years) was followed from January 2022 to January 2023. Cytokine and antibody levels were measured before vaccination, one month after the first dose, and four months after the second dose using ELISA and CRP assays. The first vaccine dose elicited a robust 330% increase in anti-spike IgG alongside significant elevations in both pro- and anti-inflammatory cytokines. Four months after the second dose, IgG levels remained 137.6% above baseline, with distinct correlations emerging between cytokine and antibody dynamics. Notably, anti-IgG/IL-10 ratios remained consistently elevated, suggesting a potential biomarker of durable immune response. These findings highlight the complex but clinically relevant relationships between cytokine regulation and antibody production, offering insights into immunological mechanisms underlying COVID-19 vaccination.

## Introduction

The global community witnessed a comprehensive scientific response to the COVID-19 pandemic, characterized by the use of many technological platforms to develop viable vaccines. The Pfizer-BioNTech BNT162b2 vaccine was one of the first approved and most widely used vaccines globally [1]. This vaccine utilizes mRNA technology, which stimulates the immune system to produce the viral spike protein, leading to integrated humoral and cellular immune responses [2].

Studies indicate that mRNA vaccines initially activate innate immunity by increasing inflammatory cytokines such as IL-1β, IL-2, IL-8, IL-17, and IFN-γ. They also activate the non-cytokine inflammatory biomarker C-reactive protein (CRP). Thus, preparing the immune system to mount an effective adaptive response [3, 4]. These cytokines exhibit distinct temporal patterns post-vaccination, with some increasing in the first days and others peaking thereafter [4, 5]. Conversely, anti-inflammatory cytokines, such as IL-10 and IL-13, play a crucial regulatory role in reducing excessive inflammation and modulating immune system activity after vaccination [6, 7].

Although multiple investigations have elucidated the impact of the BNT162b2 vaccination on immune cell activation and antibody synthesis, the correlation between pro- and anti-inflammatory cytokine dynamics and anti-spike IgG levels remains ambiguous, especially in healthy young individuals. Comprehending the interplay among these immunological indicators is essential for evaluating the quality and durability of the immune response. From this standpoint, this study aims to evaluate changes in the inflammatory cytokines IL-1β, IL-2, IL-8, IL-17 and IFN-γ, and the anti-inflammatory cytokines IL-10 and IL-13, in addition to levels of anti-spik IgG, as well as to analyze the relationship between these immune changes after receiving the Pfizer-BNT162b2 mRNA vaccine in a group of healthy young people.

## Materials and methods

### Study design and participants

This prospective cohort study was conducted at the Postgraduate Research Laboratory, College of Medicine, University of Diyala, in Baqubah, Iraq, between January 2022 and January 2023. The authors interviewed each participant and explained the study’s objective and protocol. A total of 460 students took part in the study. To ensure the reliability of our findings, we rigorously monitored participants through weekly follow-ups for 4 months, during which we assessed complete blood counts (CBC) and C-reactive protein (CRP) levels. This allowed us to detect and exclude participants with any signs of inflammation or infection, maintaining the integrity of the cohort and ensuring that the observed cytokine responses were specifically related to vaccination.

Before their participation, they underwent IgG/IgM testing using VIDAS® SARS-CoV-2 IgG & IgM (Specificity 100%, Sensitivity 99.6%, and Sensitivity) from bioMérieux company/FRANCH. To ensure that they were not previously infected or vaccinated. Among the 460 students initially screened before the study, after confirmatory testing to exclude prior SARS-CoV-2 infection or COVID-19 vaccination, 180 participants were identified as previously uninfected and unvaccinated. These individuals consented to participate in the current study and to receive two doses of the Pfizer-BNT162b2 mRNA vaccine under our supervision and follow-up, with their health monitored throughout the study period. Pro- and anti-inflammatory cytokines were assessed before vaccination, 1 month after the second dose, and 4 months after the second dose. Informed consent was obtained from the participants to collect blood samples.

### Infection monitoring during the study period

To ensure the exclusion of asymptomatic SARS-CoV-2 infection during the follow-up period, all participants underwent continuous assessment of their immune and overall health status after vaccination. Anti-nucleocapsid protein IgG antibodies were checked using the SARS-CoV-2 Nucleocapsid Protein IgG ELISA qualitative kit (Elabscience®) at post-vaccination follow-up points to detect any new natural infections that could not be caused by the Pfizer-BNT162b2 spike protein-only vaccine.

In addition, participants were followed up weekly through structured post-class meetings, during which they were clinically assessed for any symptoms consistent with COVID-19 infection, including fever, runny nose, fatigue, lethargy, headache, and increased sleepiness. Weekly blood samples were also collected in EDTA tubes for complete blood count (CBC) testing to monitor any hematological changes that might indicate infection. This integrated system of immunological, clinical, and laboratory monitoring made it possible to rule out the occurrence of new infections during the study period and to ensure that the measured immune responses were related only to vaccination without the influence of natural infection.

### Inclusion and exclusion criteria

The eligible participants were adults aged 18–23 years, all genders, in general good health, with no history of acute (within 2 weeks) or chronic pathological conditions. Participants with a history of COVID-19 infection or who received the COVID-19 vaccine or had autoimmune diseases, e.g., systemic lupus erythematosus, and pregnant women were excluded from the study.

### Vaccination of the participants

The Pfizer-BioNTech COVID-19 vaccine (Comirnaty® BNT162b2) is recommended in our community. It is an mRNA vaccine developed by Pfizer, Inc. (USA) and BioNTech SE (Germany). The primary vaccination series consisted of 2 doses, administered 21 days apart, each containing 0.3 mL of the mRNA vaccine. The vaccine is supplied as a preservative-free frozen suspension (0.45 mL) in multi dose vials. The reconstitution protocol involved adding 1.8 mL of sterile 0.9% sodium chloride solution to each vial. Post reconstitution, each vial provides six standard doses. Participants were asked to report any signs or symptoms that developed after vaccination, including pain at the injection site, fever, fatigue, headache, and excessive sleepiness.

### Determination of biomarkers

Blood samples were obtained from all participants before vaccination and at 1 and 4 months’ post-vaccination. The sera were separated by centrifugation at 1,000 × g for 10 min.

Anti-spike IgG antibodies were measured using the EPITOPE DIAGNOSTIC, INC (EDI) Quantitative SARS-CoV-2 Spike Protein IgG ELISA Kit, USA, according to the manufacturer’s instructions. This assay was used to assess the quality and quantity of the anti-spike IgG response.

Pro-inflammatory cytokines, including IL-1β, IL-2, IL-8, IL-17, IFN-γ, and non-cytokine inflammatory biomarker CRP, as well as anti-inflammatory cytokines, including IL-10, and IL-13, were measured as biomarkers of the immune response. The interleukins (IL-1β, IL-2, IL-8, IL-10, IL-13, and IL-17) were quantified using ELISA kits from ELK Biotechnology Co. Ltd., Wuhan, China. The detection ranges were as follows: IL-1β, 7.82–500 pg/mL; IL-10, 0.15–800 pg/mL; IL-2, IL-8, IL-13, and IL-17, 15.63–1,000 pg/mL.

CRP levels were measured using a Cobas C-111 analyzer (Roche, Germany) according to the manufacturer’s instructions.

### Statistical analysis

The results are presented as numbers, percentages, medians, and interquartile ranges (Q1 and Q3). The significant differences between the data before and after vaccination were determined via Wilcoxon’s paired signed rank test. The correlations between the included variables were determined via a two-tailed Spearman’s correlation test. All statistical analyses were performed using IBM SPSS Statistics for Windows (version 26, IBM Corp., Chicago). A p-value of ≤0.05 is the cutoff for a significant value.

## Results

A total of 180 healthy subjects (80 males and 100 females) with a median (interquartile range) age of 21 years (20 and 21.5) and a body mass index of 25.7 kg/m2 (24.3 and 26.6) were included.

The immune response to the first vaccination dose is characterized by significant increases in both pro-inflammatory and inflammatory biomarkers, as indicated in Table 1. Relative to the pre-vaccination baseline (0.21), the anti-spike IgG level exhibited an approximately 330-fold increase at 1 month post-vaccination, reaching 69.3. C-reactive protein showed a 14.6-fold increase. The concentrations of the pro-inflammatory cytokines IL-1β, IL-2, IL-8, and IL-17 increased by 59.7-fold, 38.6-fold, 38.2-fold, and 35.3-fold, respectively. The anti-inflammatory markers IL-10, IL-13, and IFN-γ increased by 52.9-fold, 33-fold, and 55.7-fold, respectively.

**TABLE 1:** Baseline data of 180 participants vaccinated with two doses of Pfizer-BNT162b2 mRNA vaccine.

Variables	Before	After 1 month	After 4 months	P1	P2	P3
Age, year	21.0 (20.0, 21.5)	​	​	​	​	​
Body mass index, kg/m^2^	24.7 (24.3, 26.6)	​	​	​	​	​
Anti-spike IgG antibody (DU/mL)	0.21 (0.08, 0.45)	69.3 (67.3, 73.4)	28.9 (25.3, 31.0)	<0.001	<0.001	<0.001
Interleukin 1β (pg/mL)	5.3 (4.4, 5.8)	316.2 (296.6, 348.2)	67.0 (60.1, 76.3)	<0.001	<0.001	<0.001
Interleukin 2 (pg/mL)	14.0 (11.3, 16.0)	540.1 (511.8, 613.3)	57.3 (51.7, 64.6)	<0.001	<0.001	<0.001
Interleukin 8 (pg/mL)	16.0 (13.2, 17.9)	611.1 (449.7, 707.2)	45.4 (35.2, 51.3)	<0.001	<0.001	<0.001
Interleukin 10 (pg/mL)	6.6 (4.9, 8.5)	349.4 (263.6, 398.6)	141.8 (109.8, 206.4)	<0.001	<0.001	<0.001
Interleukin 13 (pg/mL)	14.6 (12.3, 17.3)	481.7 (406.1, 515.2)	199.3 (117.0, 210.8)	<0.001	<0.001	<0.001
Interleukin 17 (pg/mL)	9.9 (8.3, 10.4)	349.6 (284.6, 427.4)	129.4 (103.3, 180.6)	<0.001	<0.001	<0.001
Interferon-γ (pg/mL)	11.7 (10.1, 12.6)	651.5 (585.7, 699.3)	208.6 (189.8, 216.6)	<0.001	<0.001	<0.001
C-reactive protein (mg/L)	0.82 (0.67, 0.91)	12.0 (10.5, 12.4)	2.4 (1.9, 2.8)	<0.001	<0.001	<0.001

The results are presented as median (Q1 and Q3). The P-value was calculated using Wilcoxon’s paired signed rank test. P1: comparison before and after 1 month, P2: comparison between before and after 4 months, P3: comparison between after one and 4 months.

Four months after the second vaccination, the immune response remained elevated: anti-spike IgG, IL-1β, IL-2, IL-8, IL-17, IL-10, IL-13, IFN-γ, and CRP exhibited increases of 136-fold, 12.6-fold, 4.1-fold, 2.8-fold, 13.1-fold, 21.5-fold, 13.7-fold, 17.8-fold, and 2.9-fold, respectively, relative to baseline. Anti-spike IgG antibodies do not correlate with pro-inflammatory or anti-inflammatory cytokines after the first vaccination dose. The anti-inflammatory cytokines were inconsistently and significantly correlated with the pro-inflammatory cytokines, with inverse correlations between IL-10 and IL-1β, IL-13 and CRP, and INF-γ and IL-8 4 months after the second vaccination, as indicated in Table 2.

**TABLE 2:** Bivariate Spearman’s correlations showed inter-relationships between pro-inflammatory, anti-inflammatory markers, and anti-immunoglobulin spike antibodies as a marker of immune response to mRNA vaccine after 1 month.

Biomarkers	IL10	IL13	INF-γ	IL1β	IL2	IL8	IL17	CRP
Anti-spike IgG	−0.231 (0.128)	0.043 (0.780)	−0.004 (0.977)	0.199 (0.189)	−0.001 (0.995)	−0.021 (0.892)	0.182 (0.23)	−0.064 (0.675)
IL10	​	−0.086 (0.574)	0.167 (0.274)	−0.307^*^ (0.040)	0.213 (0.160)	−0.091 (0.552)	−0.190 (0.211)	−0.050 (0.744)
IL13	​	​	−0.041 (0.787)	−0.081 (0.599)	0.207 (0.172)	−0.028 (0.855)	−0.268 (0.072)	0.328^*^ (0.028)
INF-γ	​	​	​	0.120 (0.432)	0.038 (0.805)	0.313^*^ (0.036)	−0.005 (0.975)	−0.149 (0.328)
IL1β	​	​	​	​	−0.244 (0.106)	0.122 (0.426)	−0.016 (0.918)	−0.096 (0.529)
IL2	​	​	​	​	​	−0.091 (0.551)	0.027 (0.861)	0.071 (0.642)
IL8	​	​	​	​	​	​	0.208 (0.171)	0.013 (0.935)
IL17	​	​	​	​	​	​	​	0.060 (0.694)

The results are presented as a correlation factor (p-value). * Significant correlation. IL: interleukin, INF: interferon, and CRP: C-reactive protein.

**FIGURE 1:**
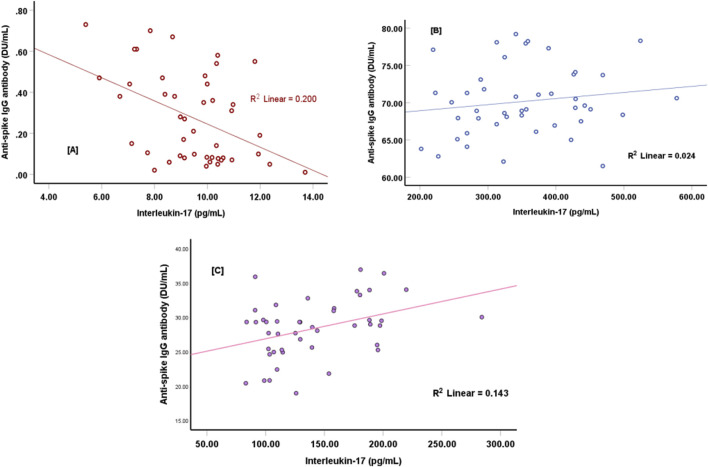
Correlations between anti-Spike-IgG antibody titers and interleukin-17 at baseline **(A)** (r = − 447, p < 0.001), after 1 month of the vaccination **(B)** (r = +0.182, p = 0.230), and after 4 months of the vaccination **(C)** (r = +0.337, p = 0.024).

The anti-spike IgG antibody level is significantly and directly correlated with the level of IL-17 (Figures 1A–C). The study demonstrated a strong positive correlation between interleukin-17 (IL-17) concentrations and anti-spike IgG levels at 1 month and 4 months after the second vaccine dose. The scatter plots in Figures 1A–C show the clustering of data points near the regression line. Figure 1A, represents the relationship between IL-17 concentrations and anti-spike IgG levels before vaccination, where the regression line indicates a negative association, as reflected by the coefficient of determination (R^2^ = 0.200). This value represents a noticeable but weak negative relationship between IL-17 concentrations and anti-spike IgG levels. Additionally, Figure 1B, illustrates the positive relationship between IL-17 concentrations and anti-spike IgG levels 1 month after receiving the two COVID-19 vaccine doses. The regression line shows a moderate positive association, supported by the coefficient of determination (R^2^ = 0.024).

Moreover, 4 months’ post-vaccination, a strong positive correlation was observed between IL-17 concentrations and anti-spike IgG levels, as indicated by the higher coefficient of determination (R^2^ = 0.143), as shown in Figure 1C.

**FIGURE 2:**
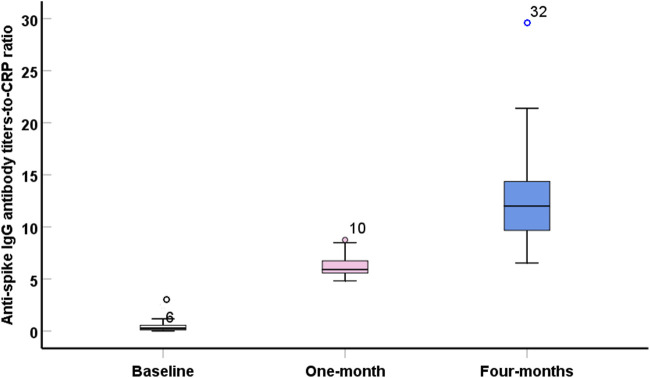
Boxplot showed significant (p < 0.001) high values of anti-spike IgG antibody titers- to – C-reactive protein ratio during the post-vaccination intervals.

**FIGURE 3:**
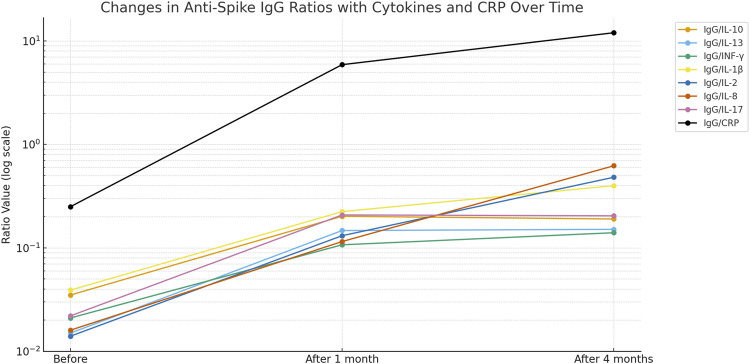
Changes in anti-Spike-IgG ratios with cytokines and CRP over time.

An inverse and significant correlation was observed with IL-10 and IL-2, IL-13 and INF-γ, and IL-1β and IL-8. In contrast, significant direct correlations were observed between IL-13 and IL-2, and between INF-γ and IL-8 (Table 3). Interestingly, the ratio of anti-spike IgG antibody level to IL10 level was significantly higher than the baseline ratio and remained elevated after 4 months following the second vaccination dose (Table 4; Figure 3). The highest ratios were observed for CRP (Figure 2) Where Before vaccination, the IgG/CRP ratio was very low because antibodies were not present. At the same time, CRP levels were normal and low. One month after vaccination, the ratio rose significantly, reflecting a considerable increase in IgG relative to CRP. Four months later, the ratio increased further as IgG continued to mature and rise, along with an increase in CRP, followed by the pro-inflammatory cytokines IL8, IL2, and IL1β.

**TABLE 3:** Bivariate Spearman’s correlations showed inter-relationships between pro-inflammatory, anti-inflammatory markers and anti-immunoglobulin spike antibodies as a marker of immune response to mRNA vaccine after 4 months.

Biomarkers	Anti-spike IgG	IL10	IL13	INF-γ	IL1β	IL2	IL8	IL17
IL10	−0.129 (0.398)	​	​	​	​	​	​	​
IL13	−0.136 (0.372)	−0.068 (0.658)	​	​	​	​	​	​
INF-γ	−0.028 (0.854)	−0.169 (0.266)	−0.326^*^ (0.029)	​	​	​	​	​
IL1β	0.103 (0.501)	0.279 (0.064)	−0.177 (0.245)	−0.099 (0.517)	​	​	​	​
IL2	−0.236 (0.118)	−0.328^*^ (0.028)	+0.300^*^ (0.046)	−0.022 (0.884)	−0.211 (0.165)	​	​	​
IL8	−0.156 (0.307)	−0.028 (0.856)	−0.060 (0.695)	+0.342^*^ (0.021)	−0.317^*^ (0.034)	0.273 (0.070)	​	​
IL17	+0.337^*^ (0.024)	−0.170 (0.264)	−0.190 (0.212)	0.027 (0.860)	0.099 (0.517)	0.073 (0.635)	−0.051 (0.739)	​
CRP	0.159 (0.298)	0.026 (0.868)	0.065 (0.671)	−0.039 (0.797)	0.081 (0.596)	0.072 (0.637)	−0.008 (0.960)	0.143 (0.350)

The results are presented as a correlation factor (p-value). * Significant correlation. IL: interleukin, INF: interferon, and CRP: C-reactive protein.

**TABLE 4:** The ratio of anti-spike IgG antibody value to the cytokines and CRP levels.

Ratios	Before	After 1 month	After 4 months	P1	P2	P3
Anti-spike IgG/IL10	0.035 (0.012–0.079)	0.202 (0.167–0.278)	0.190 (0.138–0.267	<0.001	<0.001	0.001
Anti-spike IgG/IL-13	0.015 (0.006–0.035)	0.147 (0.134–0.176)	0.151 (0.127–0.230)	<0.001	<0.001	0.029
Anti-spike IgG/INF-γ	0.021 (0.007–0.038)	0.107 (0.099–0.121)	0.140 (0.126–0.161)	<0.001	<0.001	0.001
Anti-spike IgG/IL1β	0.039 (0.016–0.096)	0.224 (0.211–0.236)	0.399 (0.360–0.514)	<0.001	<0.001	0.001
Anti-spike IgG/IL2	0.014 (0.006–0.039)	0.131 (0.116–0.140)	0.481 (0.410–0.557)	<0.001	<0.001	0.001
Anti-spike IgG/IL-8	0.016 (0.005–0.029)	0.115 (0.099–0.158)	0.621 (0.527–0.818)	<0.001	<0.001	0.001
Anti-spike IgG/IL17	0.022 (0.008–0.050)	0.208 (0.166–0.244)	0.204 (0.172–0.247)	<0.001	<0.001	0.329
Anti-spike IgG/CRP	0.250 (0.110–0.561)	5.902 (5.560–6.754)	12.008 (9.668–14.479)	<0.001	<0.001	<0.001

The results are presented as median (interquartile Q1, Q3). The P-value was calculated using Wilcoxon’s paired signed rank test.

## Discussion

The findings of the present study demonstrated that, after 1 month of immunization, both cytokine and anti-spike IgG antibody levels were higher and tended to decrease. The immune response to the Pfizer vaccine develops progressively with each dose. According to studies of all genders after the first dose, there is a measurable increase in antibody levels (IgG antibodies specific to the spike protein), providing partial immunity, primarily against severe disease [8]. This initial response usually peaks approximately 2–3 weeks after the first dose. Following the second dose, there was a significant increase in neutralizing antibody and IgG levels, with peak immunity occurring approximately 7–14 days after the second dose [9]. Additionally, the second dose triggers robust activation of T cells, which are crucial for long-term immunity and immune memory [10]. Over the next 3–6 months, antibody levels gradually decline, which is normal. Still, memory B cells and T cells continue to offer protection against severe illness. Pro-inflammatory and anti-inflammatory markers do not significantly correlate with anti-spike IgG antibodies after 1-month post-immunization; however, 4 months after immunization, there is a significant positive correlation between anti-spike IgG antibodies and IL-17. The relationship between IL-17 and anti-spike IgG suggests that activation of the Th17 pathway may contribute to enhanced humoral response and increased antibody production against the sclerosing antigen. This can be explained by IL-17 supporting an inflammatory environment conducive to B cell maturation and activation via helper T-B signaling, in addition to its role in stimulating cytokines and chemokines that promote B cell differentiation and transformation to produce higher levels of IgG [11]. A study measuring IL-17 levels in the saliva of Pfizer vaccine recipients found a significant increase after two doses, without causing an excessive cytokine storm [12], which is consistent with our current study. Therefore, IL-17 activation is not necessarily harmful; it may be part of the environment conducive to B-cell maturation. Furthermore, another study on the role of the Th17 response suggested that IL-17 (produced by Th17 cells) may contribute to the severe inflammatory response in COVID-19 infection [13].

Therefore, it can be argued that IL-17 may play a role in stimulating the immune response, not just in harmful inflammation. The cytokine storm that follows COVID-19 infection is comparable to the picture of elevated pro-inflammatory and anti-inflammatory cytokine levels observed after COVID-19 immunization, albeit to a lesser extent, and is linked to a sharp rise in anti-spike IgG antibody titers [14]. A higher level of IL-1β indicates a response to vaccination, as immunization failure may occur in patients who use IL-1 inhibitors [15]. IL-2 is a pro-inflammatory cytokine that stimulates T cells, particularly CD8^+^ T cells, thereby playing an essential role in antibody production. Participants in the current study exhibited a significant response to IL-2, thereby enhancing the production of anti-spike IgG antibodies. This conclusion aligns with the findings of Alhajjat et al. (2023) [16]. Despite showing no significant correlation with anti-spike IgG antibodies, interleukin-8 levels were substantially elevated, suggesting an enhanced inflammatory response. According to a recent study, post-acute COVID-19 vaccination syndrome is associated with higher levels of the pro-inflammatory cytokine IL-8 [17]. According to the cytokine profile of COVID-19 patients, those with longer COVID-19 disease exhibited higher IL-17 levels [18].

Consequently, persistent immunization may be indicated by a greater level of IL-17 and its association with anti-spike IgG antibodies. A noticeably elevated level of IL-10, which is typically reduced in long-term chronic COVID-19 infection, supports this hypothesis [18]. A significant association with IL-13 was observed 1 month after immunization. No significant correlation was found after 4 months, indicating that changes in C-reactive protein levels mirrored the profile seen in long-term COVID-19 syndrome, which tends to return to normal levels over time [19].

According to these findings, elevated CRP levels during the first month after vaccination may reflect an enhanced post-vaccination immune response. In addition, the positive correlations between CRP and the ratios of anti-spike IgG antibodies with each pro- or anti-inflammatory biomarker support this assumption. The anti-spike IgG antibody level is elevated by more than 10 DU/mL, indicating a persistent positive immune response for 4 months, which explains why we used the anti-spike IgG-to-other-cytokine ratio, which declined briskly within 4 months [20].

The ratio of anti-spike IgG antibody levels to cytokine levels could serve as a new, valuable parameter for assessing the positive immune response, with cytokine levels normalized to baseline. Table 4; Figure 3 show a greater ratio of anti-spike IgG antibodies to CRP after 4 months, suggesting a more pronounced humoral-to-inflammatory balance. Anti-inflammatory markers, including IL-10, IL-13, and IFN-γ, are decreased in severe COVID-19 infection. Their dynamic changes can determine the degree of protection against the lung damage induced by SARS [21, 22].

Therefore, Higher levels of anti-inflammatory biomarkers were observed; however, these findings reflect an association only and do not imply a protective effect or enhanced immunization. The strength of this study lies in the simultaneous assessment of pro- and anti-inflammatory cytokines at different time points. Another important finding is that The ratio of anti-spike IgG to CRP may serve as an indicator of changes in humoral and inflammatory status over time following vaccination, although its relevance to immunization outcomes remains uncertain. The limitations of this study include its small sample size and the exclusion of individuals with risk factors, e.g., diabetes mellitus, to illustrate differences in immune responses between healthy individuals and those at risk of contracting COVID-19.

## Conclusion

The present findings suggest that the pattern of cytokine alterations post-vaccination influences the potency and longevity of the humoral response. The IgG/IL-17 ratio may serve as a viable exploratory biomarker for assessing short- and medium-term vaccination efficacy, functioning as a hypothesis-generating indication rather than a validated clinical metric. The results demonstrate that immune response dynamics to the Pfizer-BNT162b2 vaccine involve coordinated inflammatory and anti-inflammatory activity alongside antibody responses.

## Data Availability

The original contributions presented in the study are included in the article/supplementary material, further inquiries can be directed to the corresponding author.
